# Screening and socioeconomic associations of dyslipidemia in young adults

**DOI:** 10.1186/s12889-019-8099-9

**Published:** 2020-01-28

**Authors:** Stephen E. Hudson, Matthew S. Feigenbaum, Nirav Patil, Elan Ding, Alex Ewing, Jennifer L. Trilk

**Affiliations:** 1 University of South Carolina-School of Medicine-Greenville (Affiliated with PRISMA Health), 607 Grove Road, Greenville, SC 29605 USA; 2 Furman University, 3300 Poinsett Highway, Greenville, SC 29613 USA

**Keywords:** Lipid, High-density lipoprotein, Low-density lipoprotein, Socioeconomic status, Cardiovascular disease

## Abstract

**Background:**

The Southern region of the United States is home to substantial populations with obesity, dyslipidemia, and hypertriglyceridemia, while also housing a large percentage of America’s minority, rural, and low socioeconomic status (SES) peoples. Adult-onset cardiovascular disease (CVD) research may be informed by investigating associations(s) between late adolescent demographic variables and lipid values. Our objective was to investigate lipid parameter associations with college-age socioeconomic status, which may improve age-specific screening algorithms for management or prevention of adult-onset CVD.

**Methods:**

Using an Analysis of Variance test and a general linear model, associations between gender, race/ethnicity, SES, and athletic participation on lipid parameters (VLDL-C, LDL-C, TG, TC, and HDL-C) were analyzed in 4423 private liberal arts college students enrolled in freshman-level wellness courses at Furman University in Greenville, SC. Comparative data were collected from an age-matched sample (National Health and Nutrition Examination Survey: NHANES 2003–2016). Our main outcomes were statistically significant relationships between any lipid values (TC, HDL-C, LDL-C, TG) and any demographic variables (gender, SES, ethnicity, athlete status).

**Results:**

Males demonstrated lower TC and LDL-C, and higher HDL-C values. HDL-C was highest in athletes. African-American students demonstrated healthier VLDL-C, TG, and HDL-C values. With similar distributions, the age-matched NHANES comparison group showed unhealthier values in nearly all categories.

**Conclusions:**

College students may have better lipid health than the general population. African-Americans may have seemingly healthier lipid values than age-matched individuals independent of athletic or college enrollment which has already been demonstrated in other studies. Future research should include SES relationships in lipid screening paradigms along with other appropriate risk factors for cardiovascular disease. Based on our comparative data, pediatric health providers and researchers may consider education as a potential protective factor against poor lipid health when considering lipid screening protocols for students.

## Background

The Center for Disease Control and Prevention lists the Southern region of the United States as a leader in cardiovascular disease (CVD) mortality [1]. The South is home to significant populations living with obesity, dyslipidemia, and hypertriglyceridemia, and houses many individuals from minority, rural, and low socioeconomic status (SES), all of which are independent risk factors for adult CVD [2]. Unfortunately, individuals with the poorest lifestyle choices are less likely to have any prior cholesterol or blood pressure testing performed [3]. An increased prevalence of unhealthy lifestyles in both low SES and minority groups [3] and the population at large [4] has continued to predispose adults toward poor cholesterol health and other comorbidities that lead to CVD. Indeed, lower socioeconomic status, lack of healthcare access and language barriers may explain many of the racial and ethnic disparities in cholesterol screening [4]. These studies among others give data to support the need for lipid management in all patients at risk for developing cardiovascular disease, which begins with routine screening early in life as described below.

Research in CVD demonstrates that atherosclerosis begins with a long asymptomatic phase beginning in early adolescence or even childhood [5]. Routine lipid screening in adults offers quantifiable data when assessing CVD risk based on measurements including low-density lipoprotein cholesterol (LDL-C), very low-density lipoprotein cholesterol (VLDL-C), triglycerides (TG), total cholesterol (TC), and high-density lipoprotein cholesterol (HDL-C). Performing lipid screenings as well as associating social determinants of health-based risk factors in younger generations may help identify and later prevent adult CVD; however, the 2016 United States Preventive Services Task Force (USPSTF) current recommendations for lipid screening in patients under 25 years old is classified with “I” for “Insufficient Evidence” [6]. Nonetheless, many pediatric clinicians still implement lipid screenings, subsequent education, and appropriate treatments for hyperlipidemia, if for no other reason than to identify patients with inherited dyslipidemia (which can occur in as many as 1 in 250 children who, if untreated, would otherwise maintain unhealthy LDL-C levels and may develop coronary artery disease sooner [7]) in accordance with the American Academy of Pediatrics (AAP) [8], the American Heart Association (AHA), the National Heart, Lung, and Blood Institute (NHLBI), and the National Lipid Association [9]. In addition, medical communities who screen their teenage patients assert that late adolescence represents an ideal age during which lipid screening can identify those at risk for atherosclerosis in adulthood from genetic and modifiable risk factors [9, 10]. Lipid screening research by the NHLBI and others demonstrate that total cholesterol, triglycerides, body mass index, and systolic blood pressure measured at ages 15–18 have accurately identified individuals at risk for adult CVD [7, 10]. Therefore, screening for poor lipid health and other CVD risk factors (including low SES and minority and ethnic heritage) during late youth or early college years (participants’ ages ranged 18–24) may help diagnose premorbid cardiovascular disease and thus help prevent future vascular complications and/or death [10, 11]. However, more information on the relationship between lipid values, SES, race/ethnicity, and lifestyle in young adults is needed to help resolve these disparate recommendations while also screening for early onset lipid disease in pediatric populations.

The purpose of this study was to investigate CVD risk factor associations in young adults to provide additional information that may potentially improve age-specific screening algorithms for prevention of adult CVD. This study is innovative because to our knowledge, no studies have been performed that compare SES/ethnicity and CVD risk factors among undergraduate college students enrolled in a private, liberal arts university as a part of a wellness graduation requirement program, while also comparing results to an age-matched comparison group from a nationally representative database, the National Health and Nutrition Examination Survey (NHANES).

## Methods

To determine whether cholesterol values vary with SES and minority status within undergraduate college students, and whether college students’ cholesterol health compares with a nationally-representative age-matched group, the investigators analyzed de-identified data from the Health Sciences department database at Furman University, a private liberal arts college in Greenville, SC. The Furman Health Sciences database is a comprehensive collection of student de-identified lipid values as well as other CVD risk factors (e.g. low SES and minority ethnic heritage) comprised of over 20 years of volunteer undergraduate student participation. The database supplies information to the Health Sciences department for conducting cardiovascular and other research. All de-identification occurred at Furman University with the Institutional Assessment and Research Department, and the de-identified data were given to the investigators for analysis. This secondary data analysis of de-identified data was approved by the Greenville Health System and Furman University Institutional Review Boards (through a cooperative agreement).

To populate this database from 2003 to 2015, data were collected voluntarily from students (age range 18–24 years) enrolled in the “Introduction to Health and Wellness” course taught within the Health Sciences department. Blood samples were collected by a certified phlebotomist who verified fasting status in each student prior to storing, secure shipment, and processing of the samples for lipid data values via a nationally accredited corporate lab service (LabCorps®). For this study, the associations of lipid parameters with ethnicity, financial need status, gender and athletic participation were analyzed. These values were selected in order to identify common demographic variables which may be used for routine health maintenance visits in the future as well, thus improving the clinical application of our study. Excluded values included exact age as many routine screening tests utilize broad categories of age ranges, and other values include major and university scholarship availability, as these data are also less likely to be available to clinicians though within a college a clinic may have access to this data.

Lipid values studied included VLDL-C, total cholesterol (TC), triglycerides (TG), Low-Density Lipoprotein Cholesterol (LDL-C) and High-Density Lipoprotein Cholesterol (HDL-C). To estimate SES, financial need status of each student was determined by family income reported while calculating the Expected Family Contribution (EFC) on individual Free Application for Federal Student Aid (FAFSA) responses from initial undergraduate applications. Other SES determinations included family size, ability to pay tuition, living expenses, and other costs of attendance. Final categories were created using these calculations which ultimately resulted in creation of three tiers, High Need (~$86,000 k household annual income), Medium Need (~$169,000 household annual income) and Low Need (~$189,000 household annual income).

All analyses for the Furman cohorts were carried out using SAS Enterprise Guide version 9.4 (SAS Institute, Cary, NC). The values of VLDL-C, TC, TG, LDL-C, and HDL-C were analyzed and presented as mean and 95% confidence intervals. An ANOVA test was used to determine independent association(s) of race, financial need status and gender on lipid parameters. Adjusted mean parameter values were obtained by using a general linear model; Tukey’s studentized adjustment was made for multiple pair-wise comparisons. *P*-values < 0.05 were considered statistically significant.

**Fig. 1:**
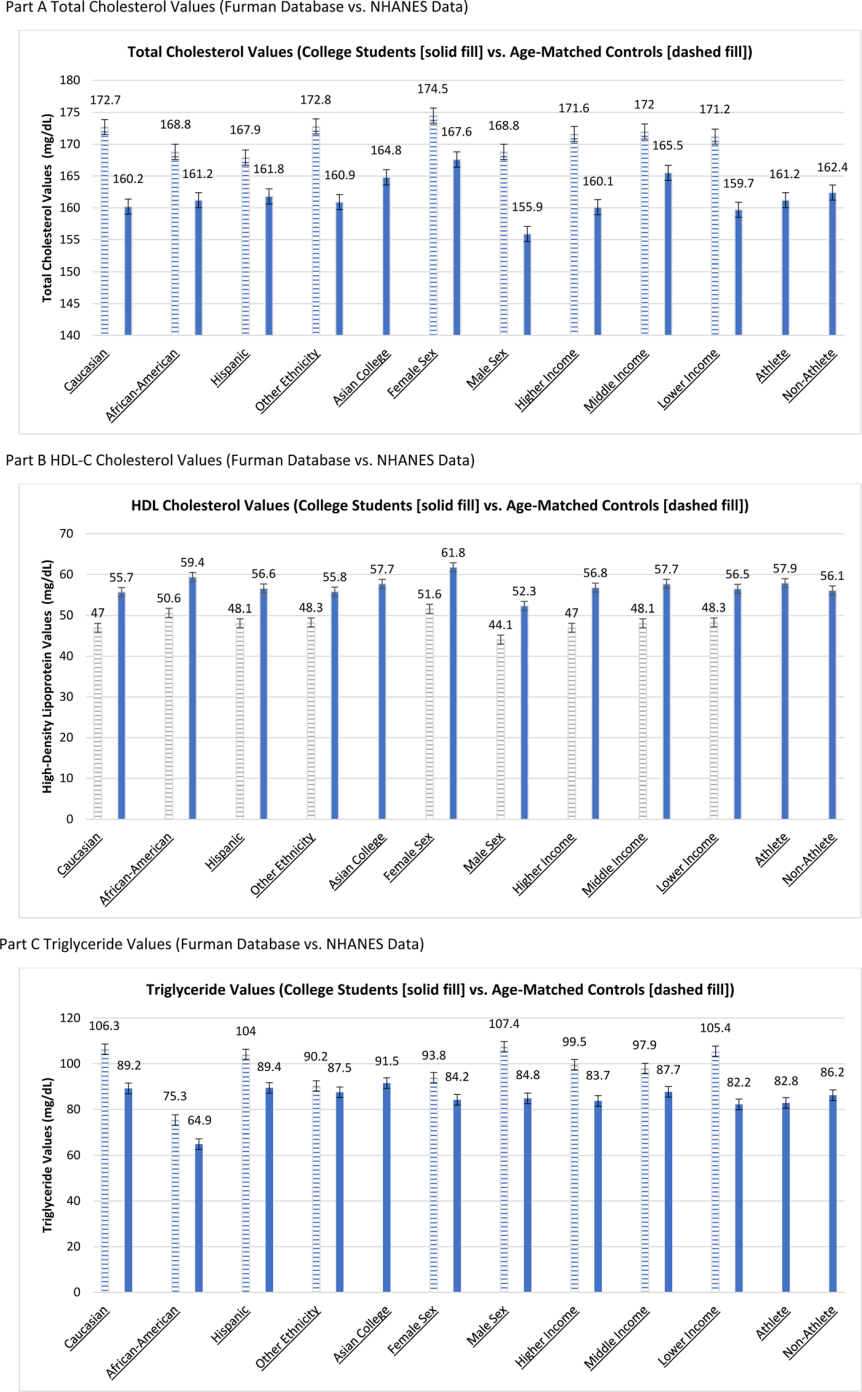
College Lipid Data versus Nationally Comparative Lipid Data (Parts A, B, and C). Part A Total Cholesterol Values (Furman Database vs. NHANES Data). Part B HDL-C Cholesterol Values (Furman Database vs. NHANES Data). Part C Triglyceride Values (Furman Database vs. NHANES Data). Legend: NHANES = National Health and Nutrition Examination Survey. Income categories defined in college (solid lines) population data as Higher (mean ~$186k), Middle (mean ~$169k), and Lower (mean ~$86k) categories using annual family income respectively (as reported by Free Application for Federal Student Aid data). For NHANES, Higher Income >$75k, Lower Income < $20k, and Middle Income between $20k and $75k

In addition, population comparisons were performed using data gathered from the National Health and Nutrition Examination Survey (NHANES) database from 2003—2016 (*N* = 4370). Using the NHANES data, similar cohorts of college-aged respondents across ethnicity, gender, and income were created. Sample weighting and analysis of the NHANES data were performed using Python (version 3.6.5). These subsets of NHANES data were weighted using the provided sample weights from each year and adjusted to fit the age ranges of the cohorts and to create nationally-representative comparisons to the Furman University data. For each of the cohorts, similar analyses were performed to provide cholesterol values and 95% confidence intervals (TC, HDL-C and triglycerides). The NHANES data was then compared to the Furman University results using the appropriate confidence intervals and the degree to which they overlap (Fig. 1). *P*-values < 0.05 were considered statistically significant.

## Results

### Furman University students

A total of 4423 college students who attended Furman University between 2003 and 15 were included in the analysis (Table 1). On average, African-American (AA) students had significantly healthier values (presented with 95% confidence intervals after each value) including lower VLDL-C at 12.7 mg/dL (11.6–13.8) vs. 17–18 mg/dL on average for all other groups (i.e. 95% CI range from 16.0–18.5 for Others and 17.1–18.2 for Caucasians). AA students also demonstrated lower triglyceride levels at 64.9 mg/dL (59.3–70.5) vs. 89–91 mg/dL on average for all other groups (i.e. 95% CI range from 86.4–92.0 for Caucasians and 81.4–93.6 for Others). Similarly, AA students showed significantly higher HDL-C levels at an average of 59.4 mg/dL (57.7–61.1) versus 55–56 mg/dL on average for all other groups (i.e. 95% CI range from 54.8–56.5 for Caucasians, and 55.8–59.7 for Others) when compared to other race/ethnicities. Male total cholesterol levels were significantly lower at 52.3 mg/dL (51.2–53.4) than in females at 61.8 mg/dL (60.7–62.8), regardless of financial need status or ethnicity. No significant associations were discovered between SES/financial need status and lipid values, except where Medium Need students displayed a slight elevation in total cholesterol at 165.5 mg/dL (161.2, 169.8) versus 159.7 (157.2, 162.2) for High Need and 160.1 (157.8, 162.4) for Low Need students.

**Table 1:** Average university student lipid parameters by race, financial need, gender, and athletic participation, 2003–15*,**

	*N* = 4423	TC^a^	TG	HDL-C^a^	VLDL-C	LDL-C
Race						
Caucasian	3697	160.2 (158.1, 162.2)	89.2 (86.4, 92.0)	55.7 (54.8, 56.5)	17.7 (17.1, 18.2)	86.4 (84.7, 88.1)
African-American	227	161.2 (157.1, 165.3)	**64.9 (59.3, 70.5)**	**59.4 (57.7, 61.1)**	**12.7 (11.6, 13.8)**	88.6 (85.3, 92.0)
Asian	169	164.8 (160.0, 169.6)	91.5 (84.9, 98.1)	57.7 (55.8, 59.7)	18.1 (16.8, 19.5)	88.6 (84.6, 92.6)
Hispanic	139	161.8 (156.6, 166.9)	89.4 (82.3, 96.5)	56.6 (54.5, 58.7)	17.7 (16.3, 19.1)	87.1 (82.9, 91.4)
Others	191	160.9 (156.5, 165.4)	87.5 (81.4, 93.6)	55.8 (54.0, 57.6)	17.3 (16.0, 18.5)	87.4 (83.7, 91.1)
Financial Need Status ^b^						
High need (~$86 k)	1387	159.7 (157.2, 162.2)	82.2 (78.7, 85.6)	56.5 (55.5, 57.5)	16.3 (15.6, 17.0)	86.4 (84.3, 88.4)
Medium need (~$169 k)	234	**165.5 (161.2, 169.8)**	87.7 (81.8, 93.6)	57.7 (56.0, 59.5)	17.3 (16.1, 18.5)	90.1 (86.5, 93.7)
Low need (~$182 k)	2802	160.1 (157.8, 162.4)	83.7 (80.5, 86.8)	56.8 (55.9, 57.8)	16.5 (15.9, 17.1)	86.4 (84.5, 88.3)
Gender						
Female	2605	**167.6 (165.0, 170.2)**	84.2 (80.6, 87.7)	**61.8 (60.7, 62.8)**	16.6 (15.9, 17.4)	**88.8 (86.6, 90.9)**
Male	1818	**155.9 (153.3, 158.6)**	84.8 (81.2, 88.5)	**52.3 (51.2, 53.4)**	16.7 (16.0, 17.5)	**86.5 (84.3, 88.7)**
Athletic Participation						
Yes	331	161.2 (157.6, 164.8)	82.8 (77.8, 87.8)	**57.9 (56.5, 59.4)**	16.3 (15.3, 17.3)	86.5 (83.4, 89.5)
No	4092	162.4 (160.3, 164.4)	86.2 (83.4, 89.1)	**56.1 (55.3, 57.0)**	17.1 (16.4, 17.6)	88.8 (87.1, 90.5)

*TC* Total Cholesterol, *TG* Triglyceride, *HDL-C* High-Density Lipoprotein, *VLDL-C* Very Low-Density Lipoprotein, *LDL-C* Low-Density Lipoprotein. All data measured in milligrams per deciliter (mg/dL). Data are presented as means followed by 95% confidence interval in parentheses

*General Linear model *p*-values. Adjustment is made for race, gender, financial need status, athletic participation and Age. *P*-Values less than 0.05 are considered significant and underlined and bolded

**Post-hoc multiple pair-wise comparison using Tukey’s adjustment

^a^No significant interaction between Race, Gender and Athletic participation ^b^ Not all students had FAFSAs (Free Application for Federal Student Aid)

### Furman University students compared to age-matched national representative group

Within the NHANES age-matched sample population, females demonstrated significantly higher HDL-C and total cholesterol levels with lower triglycerides (Table 2). African-Americans demonstrated significantly lower triglycerides. Overall, Furman students demonstrated healthier lipid averages than the NHANES age-matched comparison group in all categories, regardless of socioeconomic background or race/ethnicity including Total Cholesterol Values (see Fig. 1 part A), HDL-C (Fig. 1 part B) and Triglyceride values (Fig. 1 part C).

**Table 2:** NHANES Lipid Data (Age-Matched Nationally Representative Population), Years 2003-16

Average 2003-16 NHANES Lipid Values by Race, Gender, and Income^b,c^					
	N: 4370	Total Cholesterol	HDL-C^a^	N: 2221	Triglyceride
Race					
Caucasian	1610	172.7 (171.1, 174.4)	47.0 (46.0, 48.0)	781	106.3 (101.1, 111.5)
African-American	1137	168.8 (167.0, 170.6)	50.6 (49.3, 51.8)	575	**75.3 (71.7, 79.0)**
Mexican	771	170.4 (168.1, 172.6)	48.1 (46.8, 49.4)	400	110.6 (103.7, 117.4)
Hispanic	333	167.9 (164.3, 171.4)	48.1 (46.2, 50.0)	181	104.0 (94.8, 113.1)
Others	519	172.8 (169.9, 175.6)	50.0 (48.3, 51.6)	284	90.2 (84.0, 96.5)
Income					
High	745	171.6 (169.3, 174.0)	47.0 (45.6, 48.5)	408	99.5 (94.1, 104.9)
Medium	1521	172.0 (170.5, 173.6)	48.1 (47.2, 49.0)	792	97.9 (93.9, 101.8)
Low	1902	171.2 (169.6, 172.9)	48.3 (47.3, 49.3)	1021	105.4 (99.6, 111.2)
Gender					
Female	2304	**174.5 (173.1, 175.9)**	**51.6 (50.8, 52.5)**	1187	**93.8 (90.2, 97.5)**
Male	2066	168.8 (167.4, 170.1)	44.1 (43.3, 44.9)	1034	107.4 (103.0, 111.9)

^a^HDL-C High-Density Lipoprotein

^b^All data measured in milligrams per deciliter (mg/dL) and are available from the National Health and Nutrition Examination Survey. Data are presented as means followed by 95% confidence interval in parentheses. As in Table 1, adjustment is made for race, gender, financial need status, athletic participation and Age. *P*-Values less than 0.05 are considered significant and underlined and bolded

^c^Post-hoc multiple pair-wise comparison using Tukey’s adjustment

## Discussion

Key findings from our young adult lipid study include the relative similarity of Furman students’ lipid data as well as healthier lipid values overall when compared with the NHANES database. Our evidence suggests that there is no clear significant difference by race, socioeconomic status, or financial need category. Our data also shows some significant differences by sex as expected as well as some healthier lipid values in HDL-C, VLDL-C, and triglycerides in African-Americans which have been described in other studies. Although African-Americans also demonstrated greater percentages of athletic involvement overall, these were independent of healthier cholesterol values (with approximately 18% AA students with university-sponsored athletic team involvement versus 7.3% total student involvement, per de-identified student enrollment records). Our study suggests that most college-age students, as expected, may likely demonstrate normal lipid values throughout adolescence. A small fraction of our database showed evidence of hypercholesterolemia per NHLBI guidelines [12], suggesting that a fraction of otherwise healthy and high-functioning young adults are at risk for developing CVD sooner than the typical college graduate. Our study’s clinical significance exists primarily in the identification of cholesterol values outside of the acceptable risk range in all categories, including LDL-C, Total Cholesterol, and Triglycerides, for a liberal arts college student population. Our study’s primary research contribution is the demographic association of certain minority statuses with changes in cholesterol values, wherein African-Americans demonstrated healthier cholesterol values in VLDL-C, triglyceride, and HDL-C subcategories. Another primary research contribution is the surprising discovery that SES status has much less impact on lipid values for our study than many adult studies suggest as above. Finally, future research may benefit profoundly from the association of university enrollment with improved lipid values, and this information may be useful to clinicians when considering risk factors for future CVD in their youth patients.

According to the American Heart Association, abnormal lipid values affect 1 in 5 adolescents and screening values for dyslipidemia (> 95%) include TC > 200 mg/dL, TG > 130 mg/dL, HDL-C < 40 mg/dL, LDL-C > 130 mg/dL, and Non-HDL-C > 145 mg/dL [13]. With regard to the higher HDL-C values in Furman college African-American students, a similar Multiple Risk Factor Intervention Trial has also shown African-American males demonstrating on average 10 mg/dL higher HDL-C levels and an inverse relationship between HDL-C and SES status in African-American men vs white men (while SES status and HDL-C was directly correlated in white men) [14]. While more research is needed in this area to confirm these findings, this may give support to the concept that African-American students who are physically active and subsequently more health-conscious are also more likely to avoid CVD than other less active ethnic or minority individuals. Regardless, additional research on which socioeconomic factors contribute to these perplexing trends in cholesterol health disparities worldwide is clearly indicated due to these findings in the current population. This should also raise awareness of the possibility of different lipid thresholds existing for various ethnicities; in other words, we cannot disprove a continued higher risk for clinical CVD at an early age for certain minority populations despite normal youth lipid screening test results.

Potential confounders for the data include variations in age (although minor) as all students were between 18 and 24 per original data from Furman Health Science department collections. The significance of some data may be secondary to small or large sample sizes in comparison with the total database, especially for medium need students in Table 1 (compared with other need categories with much larger samples). When comparing Furman data to national data, possible confounding variables include availability of wellness resources and education. Students enrolled at a private liberal arts school likely exhibit a component of wellness via self-selection to education programs including Furman that encourage a healthy and wellness-focused predisposition that may be less available in other populations.

Previous studies in cholesterol research demonstrate that low socioeconomic status (SES) and minority ethnicity are established risk factors for poor cholesterol health, which is impacted by both quantifiable and nonquantifiable determinants [15–21]. Quantifiable risk factors are cholesterol values (including non-fasting triglyceride levels), hemoglobin A1c levels, smoke exposure [22], family history of stroke, diabetes, or obesity (specifically for African-American youths) [23], and [from highest to lowest odds ratios] single-living, non-white status, low income, and low education [24]. Nonquantifiable risk factors are much broader and include language barriers and lack of healthcare access [4], low and medium educational levels [17], having a negative affect in familial interactions [25], and poor health promotion behaviors including low knowledge of personal risk indicators for CVD [26, 27]. Minority status also is recognized as an independent risk factor for high blood pressure, heart attack and stroke [28], lower rates of moderate exercise [29], decreased cholesterol medication adherence [30], lower likelihood of prior lipid screening among children with stroke[s] [31], increased LDL-C, BP, and A1c among veterans [32], and even increased all-cause mortality [33].

One limitation of our study may exist as more Caucasian students attend Furman than other average university populations; however, our large sample size allowed us to include students from diverse socioeconomic and minority backgrounds with strong statistical associations within each group and lipid subcategory. We also recognize some discrepancies exist inherent within comparisons between the NHANES data and our Furman University data, including the assumption that our high financial need category in Furman’s database is comparable to the NHANES low income category (and vice versa). However, we have included the various salaries for each group in our data tables below to avoid confusion in SES classification. A strength of our study is the detailed comparison of various types of students within the Furman database such as athlete and non-athlete status and the inclusion of several levels of socioeconomic status, which is often absent from similar lipid studies. Another strength is the comparison of our original data with an age-matched control database from NHANES, which allows for unique verification of cholesterol trends between distinct groups of youths. As above, other confounders/limitations potentially include variations in age and the lack of further demographic data to delineate how Furman students differ from the NHANES data including access to wellness curriculum and physical activity resources which are essential to maintain healthy biomarkers throughout young adulthood.

As described above, adult-onset cardiovascular disease continues to be identified (and treated) by clinicians worldwide using routine lipid screening protocols in adolescence and beyond. However, even with current technological advancements in cardiovascular imaging and updated lipid screening recommendations, less is known about how to identify a younger and more insidious version of lipid disease that exists with little to no clinical symptoms in adolescence. To complicate future research in lipid screening even further, other lipid study efforts demonstrate that several nonnumeric factors including psychosocial, social, and family of origin characteristics are also related to lipid health, and that healthy behaviors in these areas maintain some degree of lifelong protection from CVD [34]. Current literature seems to lack enough well-described data or demographic associations to fully circumscribe the bulk of youth lipid disease as compared with CVD in adult populations. In accordance with the American Academy of Pediatrics and National Heart Lung and Blood Institute guidelines as above, we encourage careful consideration of lipid screening for any child during their preadolescent (ages 9–11) and adolescent (ages 17–21) primary care/health maintenance encounters, especially those with significant family history of lipid disorders or other comorbidities including obesity or high blood pressure. Otherwise delayed diagnosis and treatment is overdue when initiated at age 35 or even beyond (per USPSTF guidelines) [35], and CVD morbidity and mortality will continue to impact these individuals for many years unless change is implemented.

Other researchers have shown that African-American ethnicity is associated with healthier lipid values, most notably the Bogalusa study demonstrating higher HDL-C and lower triglycerides in African-Americans vs whites [36]. A more recent study by Sumner et al. also demonstrated similar findings and also mentions that African-Americans have higher rates of cardiovascular disease and diabetes but paradoxically are diagnosed less often with metabolic syndrome. This is likely due to the requirement that low HDL-C and high triglycerides make up two-fifths of the criteria for diagnosing metabolic syndrome (along with central obesity, hypertension, and fasting hyperglycemia), which African-Americans are less likely to have despite worse disease outcomes [37]. Thus the data our study unearthed likely represents a similar relationship and does not necessarily represent cardioprotective effects from seemingly healthier lipid values, although more research in the age group from our study is needed to verify these trends as compared with adults.

Finally, our study findings concur with the need for broader cholesterol screening as described above, as literature previously has also suggested that further advances in treating cardiovascular disease via lipid management may be realized better with broader insurance coverage, simplified cardiac risk assessments and improved access to culturally and linguistically appropriate healthcare [4]. In addition to our findings of healthier lipid values in the college database versus the national sample database, there may be an inherent association between higher SES and educational attainment, as even beginning university students are often expected to pay high tuition rates. As described in Kenik et al., these and other similar healthy lifestyle practices as well as routine health maintenance visits likely hold the greatest promise for improving cardiovascular disease screening and treatment for vulnerable populations.

## Conclusions

College-age socioeconomic status has little impact on lipid parameters within our study of private liberal arts students, but college enrollment may represent a marker for healthier lipid values when compared with age-matched individuals (from the NHANES database). Furthermore, while many pediatric and cardiovascular-specific lipid screening algorithms (AAP, NHLBI) encourage primary care providers to routinely assess lipid health in patients of all backgrounds and risk factors, many young individuals at risk for future cardiovascular disease may be missed if non-pediatric screening tools (USPSTF, etc.) are used as these models do not recommend routine adolescent lipid screening.

Finally, we hope our findings encourage other universities to adopt similar practices as Furman’s Health Sciences Department in lipid screening, in conjunction with the practical application of other wellness techniques to encourage youth health. Our data suggest that private liberal arts college students may have less CVD risk than age-matched individuals, yet some students may demonstrate significant morbidity if they remain untreated until visiting a primary care provider as a middle-aged adult. We hope to further expand this research project to help Furman University and other schools implement a similar model to predict future cardiovascular health issues in college students with immediate research-based data according to their SES/ethnicity-associated predispositions.

## Data Availability

The data that support the findings of this study are available from Furman University but restrictions apply to the availability of these data, which were used under license for the current study, and so are not publicly available. Data are however available from the authors upon reasonable request and with permission of Furman University (along with consent from collaborating partners with USC-School of Medicine-Greenville).

## Abbreviations

CVD: Cardiovascular disease
HDL-C: High-density lipoprotein cholesterol
LDL-C: Low-density lipoprotein cholesterol
NHANES: National Health and Nutrition Examination Survey
SES: Socioeconomic status
TG: Triglyceride
VLDL-C: Very low-density lipoprotein cholesterol
